# Fixation of periprosthetic or osteoporotic distal femoral fractures with locking plates: a pilot randomised controlled trial

**DOI:** 10.1007/s00264-018-4061-1

**Published:** 2018-07-25

**Authors:** Nikolaos K. Kanakaris, Oghofori Obakponovwe, Matija Krkovic, Matt L. Costa, David Shaw, Khitish R. Mohanty, Robert M. West, Peter V. Giannoudis

**Affiliations:** 1 Leeds Teaching Hospitals NHS Trust, Leeds General Infirmary, Clarendon Wing Level D, Leeds, West Yorkshire LS13EX UK; 2 Academic Department of Trauma and Orthopaedic, School of Medicine, University of Leeds, Leeds General Infirmary, Clarendon Wing Level D, Leeds, West Yorkshire LS13EX UK; 3 Addenbrooke’s Hospital Cambridge University Hospital NHS Foundation Trust, Hills Road, Cambridge, CB2 0QQ UK; 4 John Radcliffe Hospital, University of Oxford, Headley Way, Headington, Oxford, OX3 9DU UK; 5 Bradford Teaching Hospitals, Duckworth Lane, Bradford, West Yorkshire BD9 6RJ UK; 6 Cardiff and Vale Hospitals, University Hospitals of Wales, Heath Park, Cardiff, Wales CF14 4XW UK; 7 Leeds Institute of Health Sciences, University of Leeds, Charles Thackrah Building, 101 Clarendon Road, Leeds, West Yorkshire LS2 9LJ UK; 8 NIHR Leeds Biomedical Research Unit, Chapel Allerton Hospital, Leeds, West Yorkshire LS7 4SA UK

**Keywords:** Femoral fracture, Locking plate, Periprosthetic fracture, Polyaxial

## Abstract

**Introduction:**

We hypothesised that the use of a polyaxial locking plate design offers the same clinical benefits as a monoaxial locking plate system following distal femoral osteoporotic/periprosthetic fracture fixation.

**Method:**

A multicentre prospective randomised pilot trial was conducted. Inclusion criteria were patients over 60 years with a displaced osteoporotic or periprosthetic distal femoral fracture. Details documented included time to union, complications, reinterventions and functional outcomes according to the Oxford knee score and EuroQol EQ-5D. Analysis of factors influencing an early fracture healing response was performed between those with clear features of radiological callus formation at three months. Statistical analysis was performed using a logistic regression model with multiple covariates assessed for each plate system (1:1 ratio) over a follow-up period of one year.

**Results:**

Forty patients (34 females) with a mean age of 77 (60–99) were recruited. Four patients deceased within the first six months. Twenty-five patients united by the six month follow-up. Six more patients progressed to union between six and nine months. Five patients developed non-union (two patients had implant failure; one in each group) and all underwent revision surgery. Malunion was evident in two cases, one with 15° of valgus (monoaxial plate), and one with 12° of recurvatum (polyaxial plate). Between the two plate systems, statistical analysis revealed no significant differences in most of the recorded parameters. Radiological features of early bone healing were present when the surgical approach was smaller (*p* = 0.015), and when a greater working length of the bridging plate was present (*p* = 0.016).

**Conclusion:**

Both plate systems demonstrated good union rates and limited implant related complications. Good reduction, mechanically sound construct and respect of the local fracture biology was more important than the particular plate design characteristics.

**Electronic supplementary material:**

The online version of this article (10.1007/s00264-018-4061-1) contains supplementary material, which is available to authorized users.

## Introduction

Distal femoral fractures account for about 7% of all the fractures of the femur. They occur less frequently than those of the proximal end of the femur by almost ten times. [1] The distal femur is defined as the distal 15 cm from the knee joint [2], including the metaphyseal area, the femoral condyles and the joint surface.

In the presence of osteoporosis, these fractures are most commonly the result of simple falls or low energy mechanisms, in contrast to high energy injuries which are seen in younger patients, often in the context of polytrauma. [1, 3] In the elderly population, some of these fractures occur in close proximity to a prosthesis (i.e. femoral component of a total knee or hip arthroplasty) with an incidence reported to range between 0.3 and 2.5% of all primary arthroplasties. [4–7] In this cohort, osteoporosis is considered a principal risk factor. Other relevant contributing factors include anterior notching of the femoral implant, rheumatoid arthritis, prolonged steroid therapy, female sex and neurological disease. [8–11]

Distal femoral fractures are complex injuries and their outcome may be associated with severe functional impairment, permanent disability, or even death. This has been attributed to the presence of comorbidities, poor bone stock and the development of complications such as infection, malunion, non-union, cardiac and thromboembolic events. [8, 12–14]

The contemporary management of distal femoral fractures has evolved towards operative fixation for the vast majority of cases, due to the benefits of early mobilisation, the availability of specially designed implants and the high morbidity associated with prolonged bed rest. [15–18] Sophisticated implants, currently available for reconstruction of these fractures, include anatomically pre-contoured locking plating and third-generation intramedullary nailing systems introduced with a retrograde technique. [8, 19–21] Such technologies not only offer the advantage of stable fixation but also versatility to accommodate reconstruction of different fracture patterns even in the presence of arthroplasty implants. [22]

Locking plating systems include a variety of plates from stainless steel or titanium alloys, of different thickness and shape, with external targeting jigs and reduction tools facilitating minimal invasive instrumentation, as well as multidirectional or fixed angle locking options at the metaphyseal areas. [23–25]

The aims of this pilot trial were:

1. To obtain adequate data which would allow us to compute the power analysis of a future pivotal trial between implants of similar design used for these indications, by comparing the use of a plate system of newer design adopting the concept of polyaxial technology and options of insertion of different screw designs at the metaphyseal bone area (Polyaxial system, Zimmer Biomet, Warsaw, IN, USA), to that of a first generation of periarticular distal femoral locking plates (LISS system, DePuy Synthes, West Chester, PA, USA)
2. To assess the feasibility of collecting a range of functional scores for elderly patients with distal femoral fractures treated surgically at a number of post-operative time points (1, 3, 6, 9 and 12 months)
3. To ascertain risk factors of compromised healing with the use of locking plates for fixation of osteoporotic/periprosthetic distal femoral fractures

## Methods

A multicentre prospective concealed randomised pilot clinical trial was conducted from December 2010 till December 2013 in four UK Centres.

With a target number of 40 recruited patients (1:1 ratio) and a loss to follow-up of 25% incorporated to the design of the study, this was considered to yield sufficient information to calculate the standard deviation of each score (Oxford Knee Score (OKS), Pain Visual Analogue Score (pain VAS), and the self-rated Health State-Visual analogue Score (HS-VAS) necessary to determine the sample size of the proposed main trial. [26, 27] Full ethical and research approval were granted in 2010 by the Research Ethics Committee, (REC reference number: 08/H0903/26), and the Research and Development (R&D) department of the hospitals (R&D reference: OR08/8597). Funding in the form of a research grant was secured from the DePuy International Limited, registered no.3319712 (DePuy part of the Johnson and Johnson family of companies) to our department.

Inclusion criteria were patients over 60 years of age, a displaced distal femoral fracture (AO/OTA 33-A1 to C3 fractures) of a patient with diagnosed osteoporosis to his/her medical history, or a Singh index [28] grade < 4 or a displaced distal femoral fracture above or below a femoral component of total knee or total hip arthroplasty (Rorabeck type 1-2 [10], or Vancouver type C fractures [29]) respectively.

Exclusion criteria were patients with major organic pathologies (dementia, severe cardiovascular, hepatic, pulmonary, neurologic, renal or known neoplastic disease scoring above 2 in the Charlson Comorbidity Index [30]), with pre-injury impaired mobility (household or non-functional ambulatory patients) or associated trauma influencing ambulation and/or rehabilitation, as well as patients with loose femoral components (as evaluated pre-operatively based on x-rays CT-scans and intra-operative screening) and fractures as a result of infection or metastatic disease (based on the medical history of the patient).

Randomisation was performed using a ballot system of 40 sealed envelopes containing either a card of a POLYAX or LISS plating systems at a 1:1 ratio. A single sealed envelope was opened post the patient’s signed informed consent and enrolment to the study from one of the investigators. All patients were blinded to the treatment assignment until completion of follow-up as well as the outcome assessors. Radiological examination at follow-up was performed without access to the subject’s case notes. For the radiological outcome, the adjudication committee consisted of three independent musculoskeletal radiologists.

According to the protocol, closed reduction and minimal invasive techniques for fracture fixation was the default strategy. In cases where this was proven to be ineffective (inability to restore adequately limb length, varus/valgus alignment, rotational reduction), an open reduction was performed. Bicortical diaphyseal fixation as well as utilisation of all distal metaphyseal screw options was applied. All procedures were carried out in a radiolucent table in a supine position and without tourniquet.

Standard local operating procedures in regard to antimicrobial (single dose of broad-spectrum antibiotics intravenously at induction) and thromboembolism prophylaxis (low molecular weight heparin subcutaneously for a month) were followed in all centres. Free range of motion was encouraged immediately after surgery. Using elbow crutches or walking frame, weight bearing-as-tolerated was advised with physiotherapy input.

Patients per protocol were assessed clinically and radiologically on recruitment and at one, three, six, nine and 12 months post-operatively, unless further reviews were clinically indicated.

The secondary objectives of this study included the exploration of average outcome scores in each group and the impact of parameters as intra-operative details (i.e. estimated blood loss, closed vs. open reduction techniques, length of incision/s (surrogate length of all incisions in cm), duration of surgery), radiological characteristics of the plate/bone constructs, the incidence of union and malunion, of hardware failure, complications, secondary interventions. Outcome scores included the Oxford knee score [31] and the EuroQol EQ-5D. [32] Functional outcomes were collected on admission (referring to pre-injury levels of function) as well as at different time points (1, 3, 6, 9 and 12 months post-op). All fractures were classified according to the AO/OTA classification system [33] as well as the Rorabeck system [10] for the periprosthetic ones, and the level of bone density using the Singh index. [28] Osseous bone healing was confirmed with radiographs (evidence of callus formation in three out of four cortices as assessed by both AP and Lateral plain x-rays) and clinically (pain free full weight bearing). In cases that plain radiographs were inconclusive of the progress of healing, a CT investigation was carried out. Delayed union was defined as failure to heal by six months from the time of surgery, whereas no-union was defined as failure to osseous healing beyond nine months. [34, 35] Malunion was defined as shortening of more than 2 cm, a varus/valgus, procurvatum/recurvatum and rotational deformity of more than 10°. [36, 37] With regard to the specific characteristics of the bone/plate construct, a number of parameters was evaluated including the working length (length of implant around the fracture unsupported by screws), plate/screw density (rate of holes to screws used at the shaft of the plate) and the plate/span width (plate length/fracture length) [23, 38–40]. These parameters were compared in between the cases that had clear evidence of fracture healing progress at three months versus those that did not, in order to potentially identify those that contribute to a faster progress of secondary healing and early callus formation. In addition, this clinical trial aimed to provide data for sample-size calculations, which would guide the design of larger pivotal multicentre studies of powered adequately to be able to demonstrate which characteristics of the different treatment methods can achieve statistical significance.

The analysis of the accumulated data was performed using a Logistic Regression of Union and Malunion on covariates that included use of either Plating system with variables comprising of age, sex, smoking status, Charlson comorbidity index, mechanism of injury, type of fracture, value of Singh osteoporosis index, period of non-weight bear, time to partial-weight bear, time to full-weight bear, complication rates, pain VAS, Quality of life score—EuroQol 5D and knee functional outcome scores. Statistical significance was set to the *p* value < 0.05. Interrater reliability between the blinded independent radiologists was also utilised using Cohen’s Kappa value. All statistical analyses were performed using Microsoft Open R version 3.2.3. In order to identify promoting factors of fracture healing, variables of interest between patients with or without evidence of healing by three months, the Student’s *t* test for continuous variables and Pearson’s chi-squared test for categorical variables was used. All of the variables of interest were considered in a logistic regression model. Every combination of the 15 main effects was considered, a total of 32,768 models. The model with the lowest value of Akaike’s Information Criterion (AIC) was selected and reported. The R package glmulti version 1.0.7 was used for this purpose. The final model was assessed using a receiver operating characteristic (ROC) curve reporting the area under the curve (AUC), sensitivity and specificity which quantify its prediction performance. The ROC curve was generated using the Epi package version 1.1.71. In regard to the analysis of the functional outcomes at different time points, individual patient trajectories (spaghetti plots) were plotted against time in order to visualise the progression of patients. Mean values by implant were calculated at each of the time points. Further, a smoothed trend was calculated over time complete with confidence interval, separately for each implant type. These were displayed on the patient trajectory plots. Functional outcomes were regressed upon time, taking time as a categorical variable (values were 0, 1, 3, 6, 9 and 12) rather than as a continuous one. This permitted non-linear time trends which can be seen to be necessary from the visualisations. To account for the clustering of functional outcome measures within patients, a random intercept for patient was included in a multi-level model. The three functional outcomes measures (Oxford Knee score, OKS; pain VAS and general health state visual analogue scale, HS-VAS) were considered in separate regression models. No fixed intercept was fitted so that values at different times reflect average values.

## Results

**Fig. 1:**
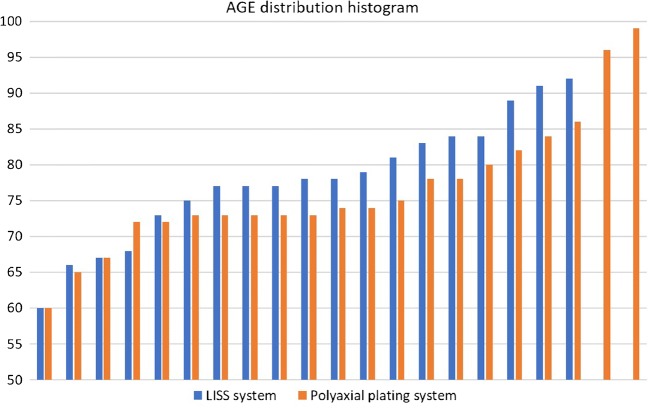
Histogram of the age distribution of the 40 recruited patients to the study, stratified per implant type

**Fig. 2:**
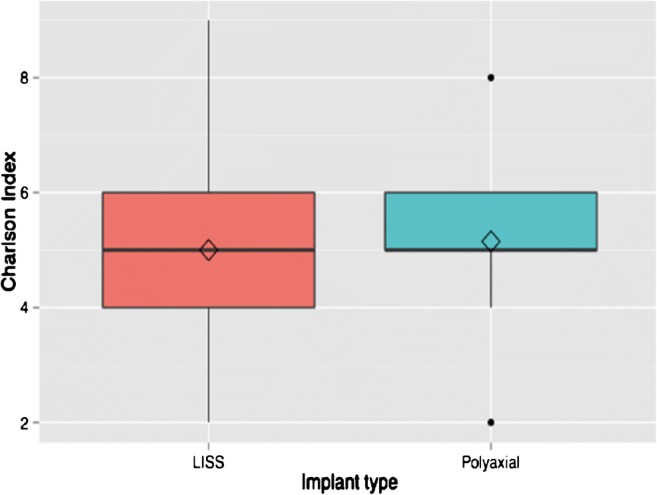
Boxplots of the Charlson comorbidity index by implant type

**Fig. 3:**
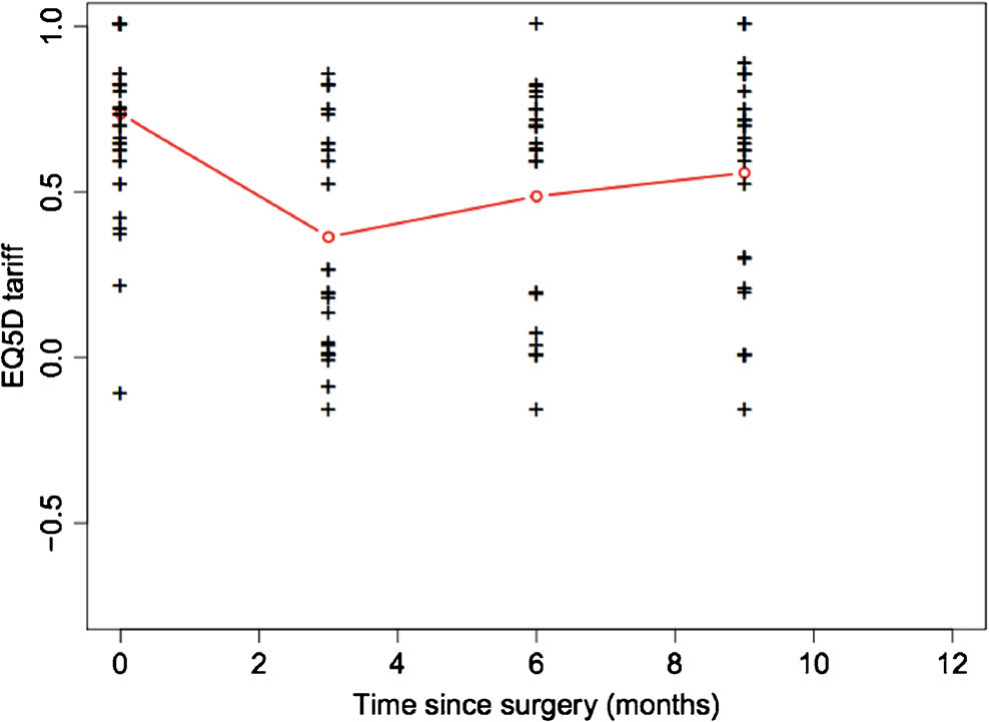
Evolution of EQ-5D tariffs over time following surgery

Between the two plating systems, there were no significant statistical differences in most of the recorded parameters (Table 1; Figs. 1, 2 and 3), including the operation time (*p* = 0.23), length of incision (*p* = 0.39), duration of hospitalisation (*p* = 0.89), fracture union at six months (*p* = 0.73), OKS score (*p* = 0.77) and EQ-5D score (*p* = 0.35), which were all comparable.

**Table 1:** Basic characteristics and comparative analysis between the two groups of locking plate fixation of distal femoral fractures

	Polyaxial plating system	LISS system	*p* value
Number of cases	21	19	
Gender ratio (F/M)	18/3	16/3	0.44
Age*	77, 76.8 (60 to 99 years)	77, 77.4 (60 to 92 years)	0.22
Side of fracture ratio (right/left)	13/8	12/7	0.82
Charlson comorbidity score*	5, 5 (2 to 8)¥	5, 5.1 (2 to 9)¥	0.42
Singh index*	2, 2.2 (1–3)	2, 2.2 (1–4)	0.46
No of periprosthetic fractures (TKA)	9 42.9%	8 42.1%	0.82
33.A2/3/B/C no, %	14 66.7%	5 26.3%	0.06
Open reductions no, %	4 19%	6 31.6%	*0.04* *
Length of incisions*	15, 15.1 (7 to 33 cm)	15, 14.8 (7 to 24 cm)	0.46
Duration of surgery*	90, 100.2 (70–192 min)	90, 100.4 (60–168 min)	0.23
Plate screw density (rate of holes to screws at the shaft of the plate)*	0.44, 0.5 (0.33 to 0.83)	0.44, 0.5 (0.25 to 0.83)	0.43
Working length*	134, 131.7 (46 to 213 cm)	133, 130.7 (40 to 227 cm)	0.77
Plate span width (plate length/fracture length)*	2.2, 2.5 (1.5 to 4.3)	2.2, 2.5 (1.3 to 7.7)	0.47
Length of stay *	19, 20.5 (10 to 43 days)	19, 20.6 (4 to 42 days)	0.83
Early signs of healing at 3 months No, %	9 42.9%	6 31.6%	0.14
Union rates at 6 months No, %	12 57.1%	13 68.4%	0.73
Union rates at 9 months No, %	15 71.4%	16 84.2%	0.63
Secondary surgeries No, %	2 9.5%	5 23.8%	0.02*
Hardware related problems No, %	1 4.8%	6 31.6%	0.002*
Malunion No, %	1 4.8%	1 5.3%	0.83
Mortality within 12 months No, %	3 14.3	1 5.3%	0.15

¥Charlson score (median, mean and range) represented as the summation of scores of the different comorbidities recorded in each of the groups as per reference 29

Italics imply statistical significant difference noted

*Median, mean, (range)

However, a statistical significant difference was noted in regard to hardware-related problems and secondary surgeries in favour of the polyaxial system, Table 1.

Analysis of factors influencing an early fracture healing response was performed in 15 cases (39.5%) versus the rest, Tables 2 and 3. On plotting the ROC curve, the selected model gave AUC = 0.881, sensitivity 82.6% and specificity 86.7%., indicating good prediction performance. There was no advantage in between the two different plating systems to this analysis, neither from factors as the demographics, comorbidities, fracture comminution, nor the ratio of cases with an intramedullary stem proximally, or percentage of filled holes. However, there was statistically significant higher ratio of early healing radiological features when the surgical approach was smaller (*p* = 0.015), and when a greater working length of the bridging plate was present (*p* = 0.016). The ratio of working length to the length of the fracture also appeared as a factor associated positively with early bone repair. As shown at Table 3, the latter did not reach statistical significance through the Wald test for its coefficient but note that the range of this ratio was wide (0.75–3.27), which means that the size of this effect can be large, indicating that this variable in the fitted model merits further investigation.

**Table 2:** Comparative analysis between distal femoral fractures managed with a locking plate as to the evidence of healing at 3 months, to identify factors influencing fracture healing

Characteristic	Not healed at 3 months	Healed at 3 months	*p* value of test
*N*	*23*	*15*	
Gender male (%)	3 (13.0)	2 (13.3)	0.999
Age*	77.52 (9.90)	76.60 (8.25)	0.767
Charlson score*	5.00 (1.04)	4.73 (1.16)	0.467
Singh’s index*	2.09 (0.73)	2.27 (0.59)	0.433
Classification type*	0.70 (0.97)	1.07 (1.03)	0.270
Second generation of plating (%)	11 (47.8)	9 (60.0)	0.687
Total incision length in centimetres*	16.74 (6.27)	12.23 (3.25)	0.015*
Problems with metalwork (%)	6 (26.1)	2 (13.3)	0.592
Bridging femoral stem*	4 (17.4)	1 (6.7)	0.642
Percentage of filled holes*	49.94 (15.3)	50.3 (15.5)	0.627
Ratio of working length to fracture length*	1.60 (0.53)	1.44 (0.45)	0.323
Working length in centimetres*	108.40 (40.72)	145.78 (46.81)	0.016*
Fracture length in centimetres*	79.27 (28.98)	102.30 (48.73)	0.108

Italics imply statistical significant difference noted

*Mean (SD)

**Table 3:** The results of the performed analysis based on the fitted logistic regression taking into confounding factors included in the model

Risk factor	Unadjusted OR (95% CI)	Adjusted OR (95% CI)	*p* value for adjusted OR
Total incision length	1.21 (1.03, 1.43)	1.35 (1.05, 1.73)	0.02*
Ratio work length by fracture length	2.11 (0.48, 9.20)	4.12 (0.66, 25.72)	0.13

Italics imply statistical significant difference noted

*p < 0.05

Following post hoc sample size calculations (the STATA® data analysis and statistical software system was used to determine the sample size of a similar appropriated powered pivotal study). With an 80% power, limiting the chance of a type II error to 20%, and a detection sensitivity of the union rates between the two equal groups of at least 5%, it was calculated that 1890 patients (945 patients in each arm) would be required.

**Fig. 4:**
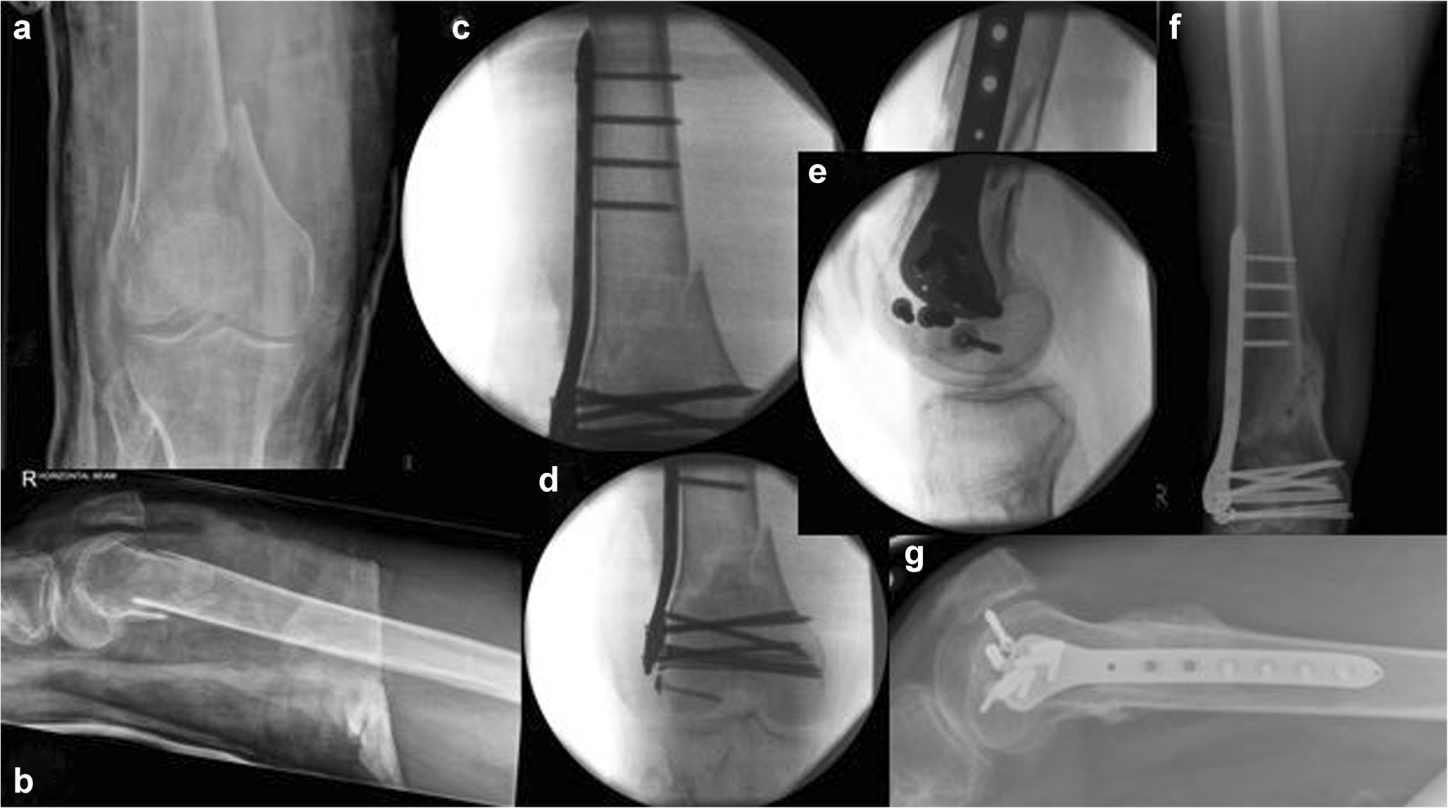
Type 33.C2 distal femoral fracture to a 67-year-old lady following a fall from standing height (**a** and **b** represent preoperative anteroposterior and lateral views of the right distal femur). Randomised to and fixed with a Polyaxial distal femoral plate and three free lag screws addressing the intra-articular extension of the fracture lines, (**c**, **d**, **e**). Uneventful fracture healing was evident to the radiological control at 6 months (**f**, **g**) and recovery of pre-injury levels of mobility, knee function, and overall health state recovery

Forty patients were recruited following informed consent based on the original design of the study. Basic characteristics of the randomly assigned groups are shown in Table 1. The groups were comparable in terms of patient characteristics (gender, age and comorbidities). Four patients deceased within the first six months of the study. Twenty-five patients united by the six month follow-up Fig. 4. Six more patients progressed to union between six and nine months. All fractures united by secondary type of healing at a mean time of 5.2 months (SD = 3 months).

**Fig. 5:**
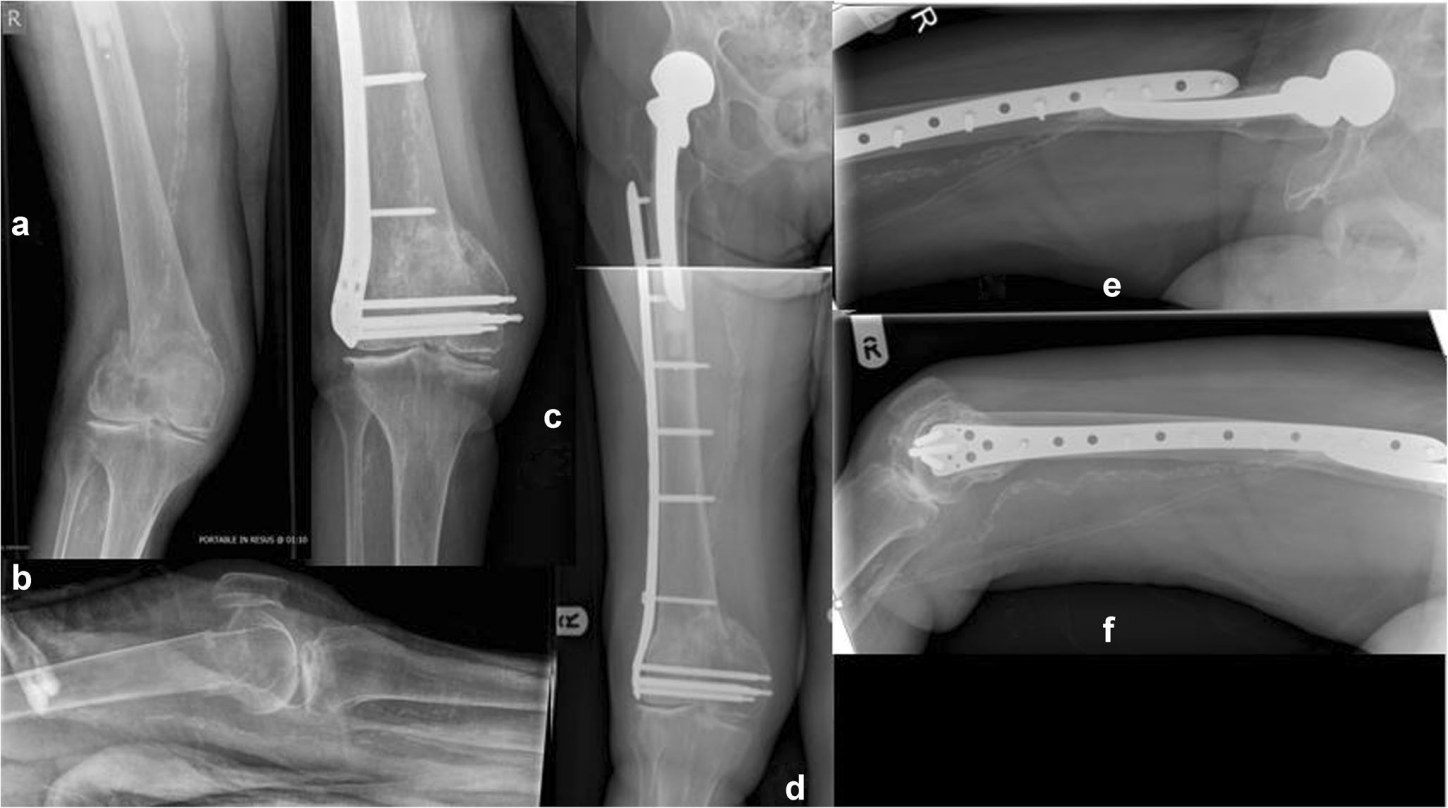
Type 33.A2 distal femoral fracture of a 92-year-old lady following a fall at her garden (**a** and **b** represent pre-operative anteroposterior and lateral views of the right distal femur). Of note, an ipsilateral cemented Thompson’s hemiarthroplasty. Randomised and treated with a LISS plating system inserted with a mini invasive technique, spanning proximally the stem of the hemiarthroplasty to neutralise an in-between implants stress riser (**d**, **e**, **f**). The fracture was noted to be fixed and was malunited in 15° of the valgus (**c** and **d**). The same patient underwent further surgery 9 months later whereas two of the metaphyseal locking screws were removed as they were found to be irritating the soft tissues over the medial femoral condyle (**c**)

Five patients developed non-union (two patients had implant failure; one in each group); all five underwent revision surgery (two underwent revision of fixation to a retrograde femoral nail; no bone grafting was used; two received bone graft augmentation without revision of the osteosynthesis; one patient underwent revision of fixation (re-plating) and bone grafting). Another two patients had a secondary procedure for removal of long screws from the distal metaphysis as they were irritating the soft tissues over the medial femoral condyle Fig. 5. Malunion was evident in two cases, one with 15° of valgus (LISS plate, Fig. 5), and one with 12° of recurvatum (polyaxial plate).

On admission, all but two patients were noted to take calcium/vitamin D tablets as well as bisphosphonates orally. During the study period, none was administered any form of medication known to affect bone healing (non-steroidal anti-inflammatory drugs, corticosteroids or anabolic agents).

**Fig. 6:**
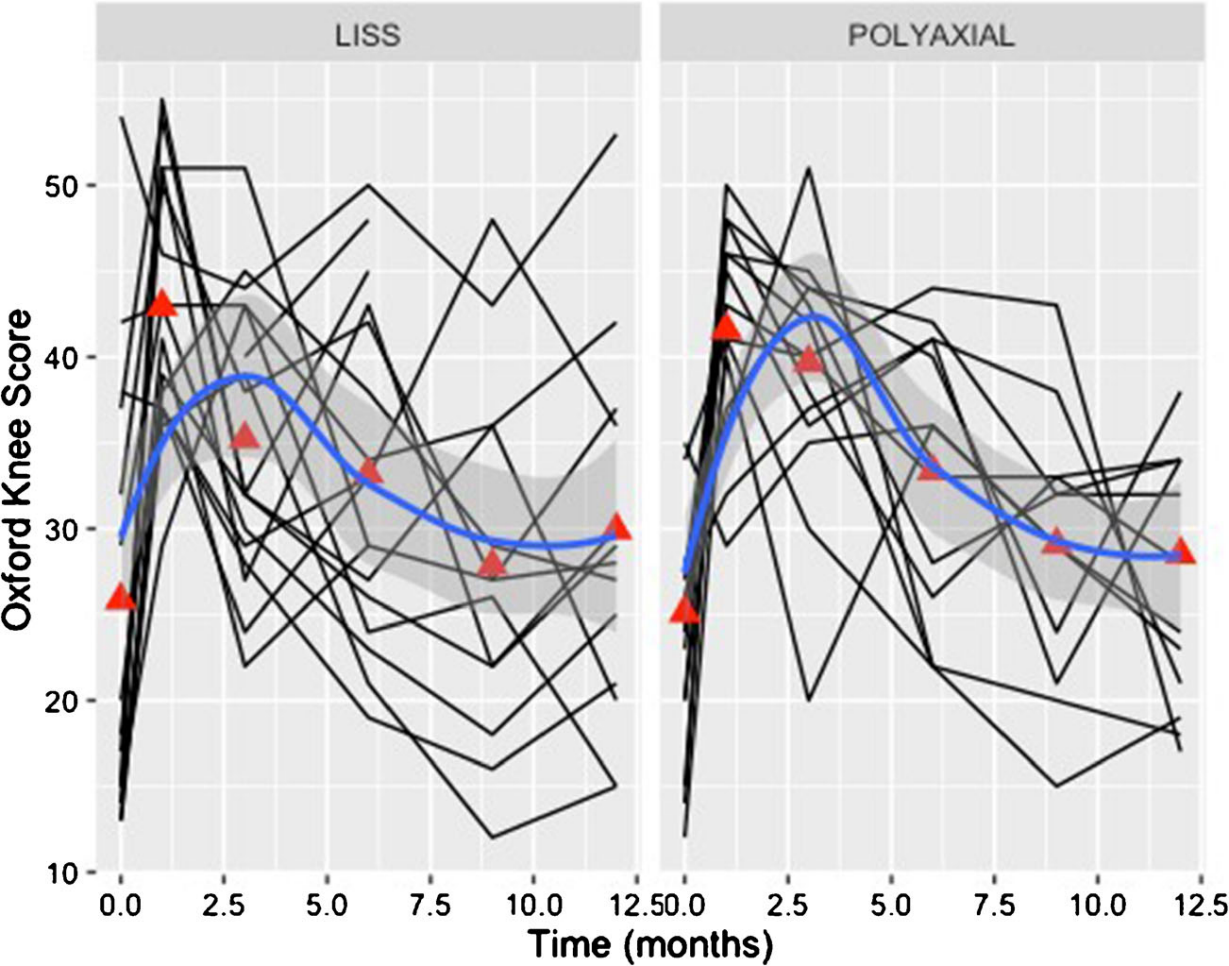
Individual patient trajectories (spaghetti plots) plotted against time demonstrating the progression of the recorded knee score (Oxford Knee Score (OKS)). Mean values, by implant, were calculated at each of the time points (red triangles—baseline, 1, 3, 6, 9, 12 months). The blue line represents a smoothed trend of the progress of the OKS measure for each implant type

**Fig. 7:**
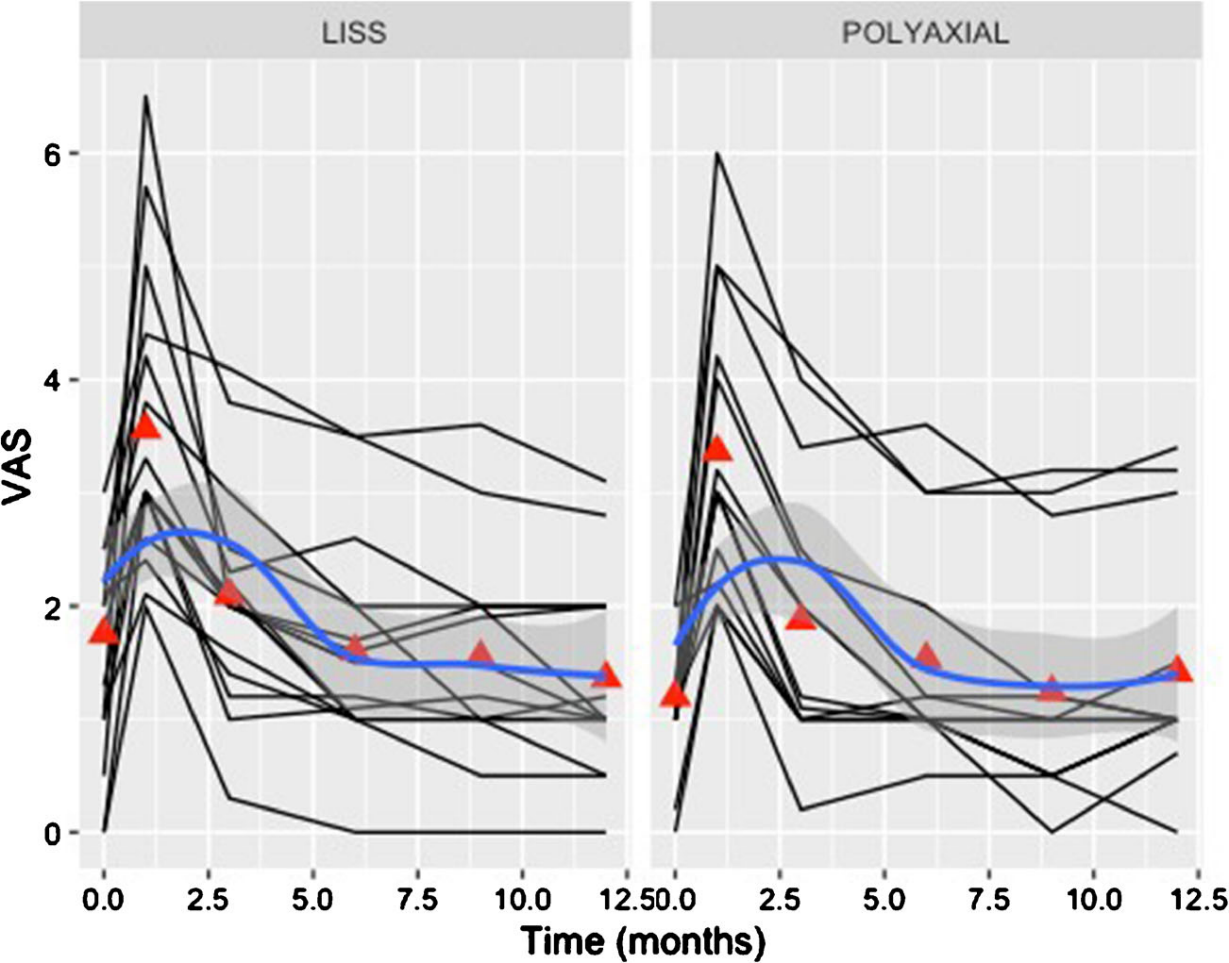
Individual patient trajectories (spaghetti plots) plotted against time demonstrating the progression of the recorded pain score as per the visual analogue scale VAS. Mean values, by implant, were calculated at each of the time points (red triangles—baseline, 1, 3, 6, 9, 12 months). The blue line represents a smoothed trend of the progress of the VAS measure for each implant type

**Fig. 8:**
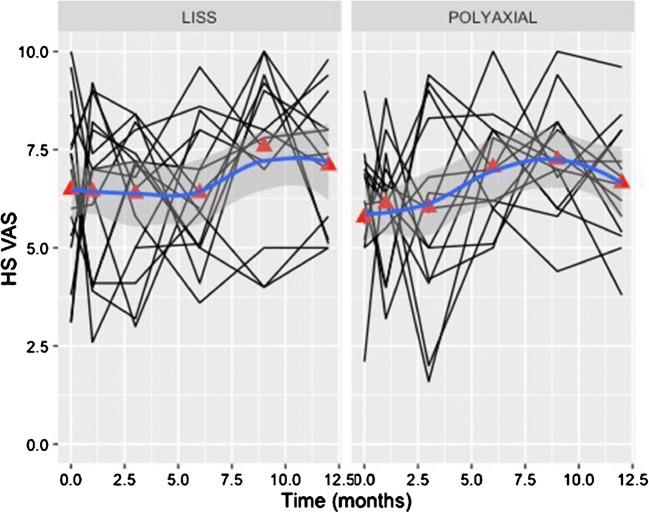
Individual patient trajectories (spaghetti plots) plotted against time demonstrating the progression of the recorded pain score as per the general health visual analogue scale HS-VAS of the EQ-5D. Mean values, by implant were calculated at each of the time points (red triangles—baseline, 1, 3, 6, 9, 12 months). The blue line represents a smoothed trend of the progress of the HS-VAS measure for each implant type

The results of the fitted regression models of the functional outcome measures (OKS, pain VAS, HS-VAS) reflecting the recovery of the patients are summarised in Table 4. The functional outcome as measured by the Oxford knee score as well as the pain levels and the general health state appeared to steadily improve following the respective progress of fracture healing and mobilisation. The majority of patients reached their pre-injury knee function (Fig. 6), and reported pain scores similar to baseline (Fig. 7) at the six months follow-up. Their general health state score (HS-VAS) appeared to continue to improve up to 12 months, (Fig. 8).

**Table 4:** A summary by their coefficients of the fitted regression models of the analysed three functional outcome measures (OKS, pain VAS, HS-VAS of EQ-5D) between the two devices

Functional outcome measure	Polyaxial vs. monoaxial implant	*p* value implant	Variance random intercept	Variance residual	Interclass correlation
OKS	+ 0.5	0.81	19.6	62.9	0.24
VAS	− 0.2	0.36	0.61	0.33	0.65
HS-VAS	− 0.3	0.36	0.28	3.14	0.08

## Discussion

In this study, stabilisation of distal femoral fractures with locking plating systems in elderly patients has been evaluated in a multicentre prospective concealed randomised pilot trial. Collection of functional and clinical outcome scores at different time points was proven to be feasible.

Both locking plating systems achieved similar union rates at comparable time frames without differences observed on the recorded intra-operative parameters, the incidence of non-union, malunion, hardware failure and functional outcomes. Whilst the polyaxial system was associated with less secondary interventions and hardware-related problems, a finding, which could be attributed to the more comprehensive plate and screw design of the polyaxial system (more screw length sizes available and options to direct screws), the nature of the herein study does not allow us to be categorical.

Noteworthy, in both systems, when the surgical approach was smaller and when a greater working length of the bridging plate was present, early radiological features of bone healing were observed. This is in agreement with previous studies highlighting the benefits of biological plate fixation and the biomechanical advantages of a balanced construct. [23, 39–44]

The data obtained in this pilot trial allowed us to compute the power analysis of a future pivotal trial between implants of similar design. However, it should be acknowledged that as the incidence of distal femoral fractures is relatively low, a large proportion of the affected elderly patients can be cognitively impaired; the mortality and lost to follow-up rates can be significant, such a large scale pivotal study will require huge resources and multiple centres worldwide. Consequently, we can conclude that such a pivotal trial is not feasible.

A contemporary consensus on the ideal plating system for the osteosynthesis of fractures of the distal femur has not been reached. A large number of clinicians prefer locking plates of fixed angle trajectories, whilst others advocate in favour of systems with variable angle options. The polyaxial plate belongs to the second generation of locking osteosynthesis systems, allowing a cone of 30° of freedom during the insertion of the meta/epiphyseal screws. The LISS plate is a first-generation locking system, with fixed angled trajectories to all its screws, at 90° to the plane of the implant. In theory, the possibility to insert locking screws in variable angles, especially at the meta/epiphyseal area, and still have a sufficiently robust angular stable construct, offers advantages to the operating surgeon. More specifically, avoiding highly comminuted areas, targeting zones of good bone stock or avoiding pre-existing implants and free screws can be particularly useful in certain clinical scenarios.

Whilst this versatility of the polyaxial plating system is considered advantageous compared to the traditional monoaxial (LISS) system, there are limited published clinical studies to demonstrate effectively this concept. Hanschen et al. [6] reported no surgical complications and all fractures of their 27 studied patients united. They went on to state that treatment with the polyaxial NCB® system (Zimmer Biomet, Warsaw, Indiana, 46581-0708, USA) demonstrated improved radiological and functional outcome in comparison to the LISS system. However, the recruited patients of that study included even young patients with distal femoral fractures following high energy trauma in contrast to our patient cohort which represents the elderly population. Moreover, the size of cohort was small, and patients’ comorbidities, rates of complications and secondary procedures were not clearly described. The present study supports the view that even in the challenging environment of osteoporotic and periprosthetic fractures, the type of surgical approach and bone-plate construct is more important than the type of plating system used.

Herrera et al. [9] carried out a systematic review of 415 periprosthetic distal femoral fractures where the patient population studied matches better to our study cohort. The authors reported an infection rate of 3%, fixation failure rate of 4% and a non-union rate of 9%, which are quite similar to our findings of infection rate at 2.5%, fixation failure at 5%, non-union at 11% (when patients that died during the period of follow-up were excluded) and mortality at 10%. Previous biomechanical and clinical studies have evaluated the use of variable angle locking plating systems for the fixation of distal femoral fractures. [5, 25, 45–47]

Wilkens et al. [46] advocated in favour of a variable angle construct, showing advantages to the load-to-failure and to the stiffness in their study based on a synthetic bone model. The analysed implants in that study were manufactured by Zimmer. However, there have been question raised in regard to the biomechanical characteristics of the variable angle systems in comparison to the fixed angled ones. [45] In contrast, the system of POLYAX® was found to have inferior performance in comparison to the LISS, especially in regard to the load-to-failure, the peak-force and stiffness behaviour of the constructs in a number of biomechanical studies. [45, 46, 48] A precise explanation of the reason that the variable angle systems withstand less axial loading forces has not been identified. One reason maybe that the bushings of the POLYAX® system that exist into the screw holes of the metaphyseal part of the plate and allow a cone of 30° of variable angle trajectories, lead to reduced load bearing performance of this system in comparison to the fixed angled plating systems.

This pilot RCT study has shortcomings, besides its small numbers and the relatively limited follow-up period of 12 months. The surgical fixations were performed by six specialist trauma surgeons. The familiarity of each of the surgeons’ with either of the two systems was not strictly controlled or matched during the phases of the trial. Thus, the learning curve of each surgeon with any of the two plating systems may have affected the recorded outcomes. Furthermore, imbalance between the two groups in terms of baseline characteristics have not been fully neutralised at the phase of randomisation. Diabetes or smoking, known risk factors of delayed fracture healing, general comorbidities, which affect the general health state, functional capacity and outcome of the patients, where also not considered. However, the general baseline characteristics of our two study groups were found to be evenly matched. By chance, more complex patterns of distal femoral fractures (33A3/33B2) were allocated to the Polyax™ group (6 vs. 2), as a result of the non-stratified randomisation process. This could have skewed the results in favour of the LISS system, as usually the complexity of the fracture pattern leads to a more technically demanding surgical procedure. Most of all, the most striking limitation of this study is its small size and the effect of a type II error. However, this trial was designed as a feasibility/pilot study. The assigned length of follow-up was considered adequate by the Ethical committee taken into consideration the acute nature of the condition under investigation (fracture healing) and the inherent difficulties and peculiarities of prolonged monitoring of elderly fracture patients. [49, 50]

Strengths of the study include its prospective randomised nature, as well as the preliminary analysis of factors influencing positively an early fracture healing response which could be considered for the design of future clinical trials. In addition, the data obtained allowed us to compute the power analysis of a future pivotal trial between implants of similar design.

Both plating systems demonstrated good union rates and limited implant related complications. A balanced bone plating construct and respect of the local fracture biology appeared to be more important than the particular plate design characteristics.

## Electronic supplementary material

ESM 1 (PNG 83 kb)

High resolution image (TIFF 543 kb)

ESM 2 (DOCX 23 kb)
