# Supplementary figures and images for: Fixation of periprosthetic or osteoporotic distal femoral fractures with locking plates: a pilot randomised controlled trial
Source: Int Orthop. 2018 Jul 25;43(5):1193–204. doi: 10.1007/s00264-018-4061-1 (PMC6470115; doi:10.1007/s00264-018-4061-1)

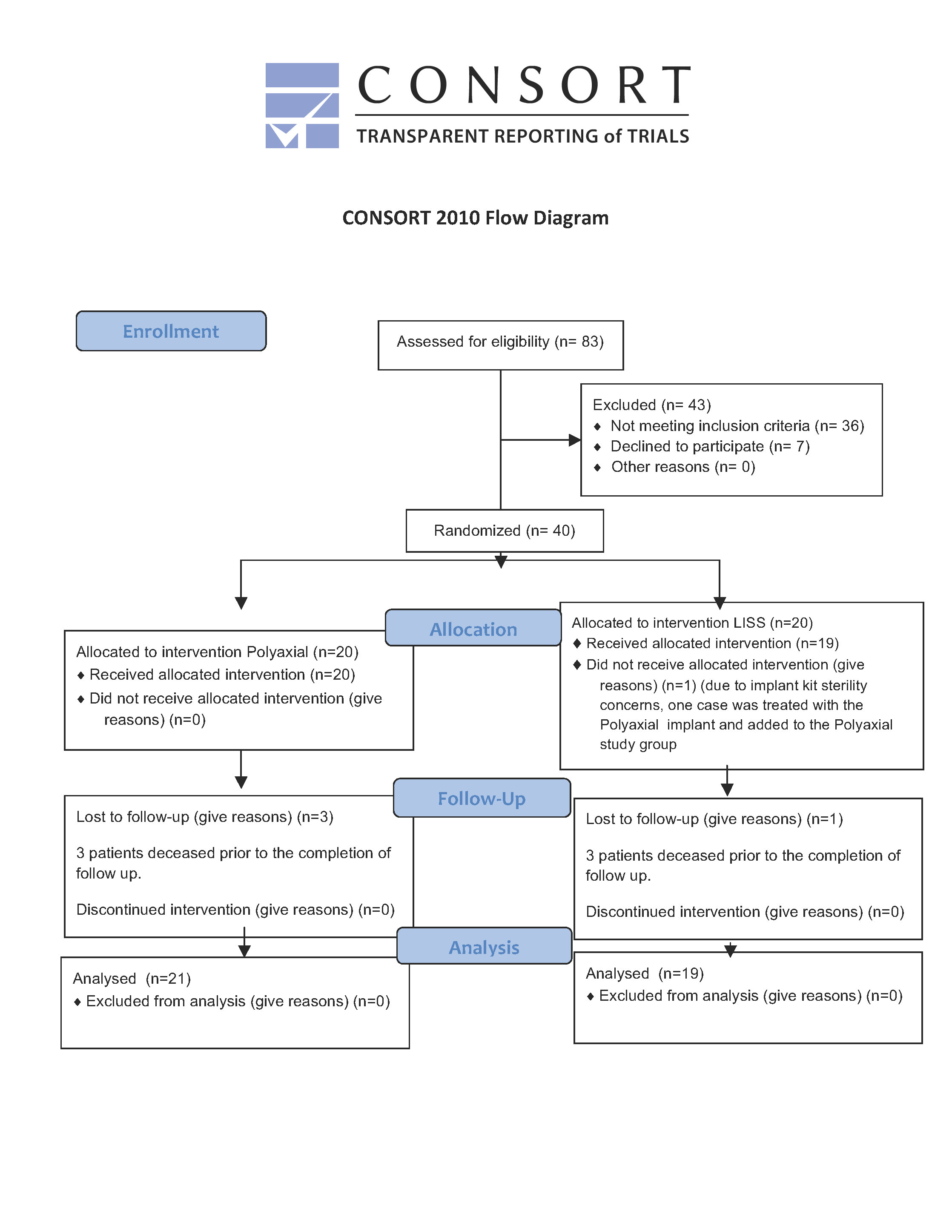

Supplement: Supplementary file 1 — (PNG 83 kb) [file 264_2018_4061_Fig9_ESM.png]

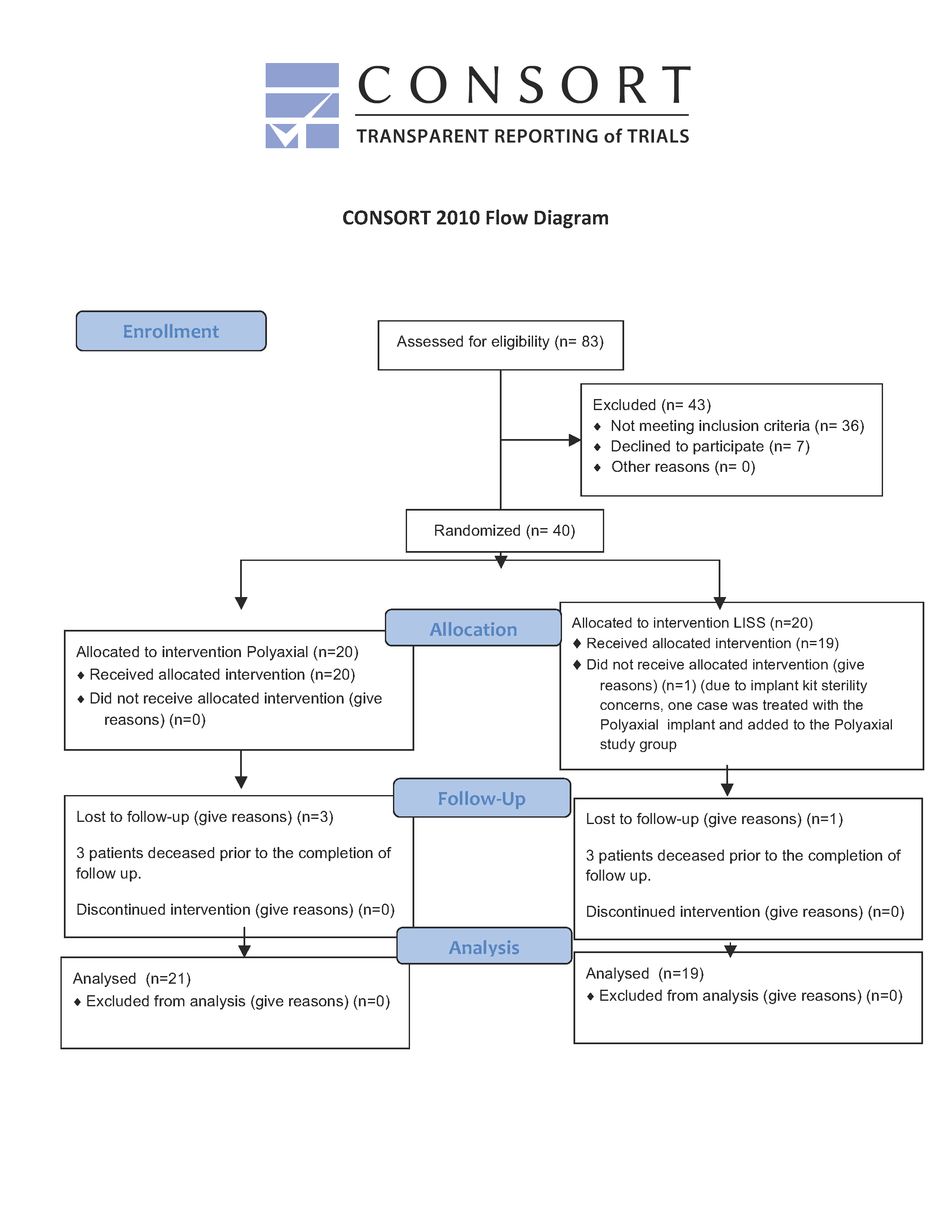

Supplement: Supplementary file 2 — High resolution image (TIFF 543 kb) [file 264_2018_4061_MOESM1_ESM.tiff]
